# Bovine intracranial neoplasia: A retrospective case series

**DOI:** 10.1177/03009858221100433

**Published:** 2022-05-31

**Authors:** Hanne Jahns, Maire C. McElroy

**Affiliations:** 1 University College Dublin, Dublin, Ireland; 2 Department of Agriculture, Food and the Marine, Celbridge, Ireland

**Keywords:** brain, cattle, immunohistochemistry, isocitrate dehydrogenase gene, neoplasia, neuropathology, OLIG2

## Abstract

This case series describes the clinical and pathological findings of intracranial neoplasms in cattle, a rare entity. Data and archived tissues from 24 intracranial tumors were reviewed and investigated by immunohistochemistry for S100, glial fibrillary acidic protein, synaptophysin, pancytokeratin, vimentin, neuron-specific enolase, oligodendrocyte transcription factor 2, and isocitrate dehydrogenase 1. Ages of affected cattle ranged from 6 months to 14 years (5.7 ± 3.6 years; mean ± SD). Predominant clinical signs were altered mental state, central vestibular dysfunction, and cerebellar incoordination. Twelve gliomas, all high grade, were the most common tumors observed: oligodendrogliomas (n = 6), astrocytomas (n = 4), and undefined gliomas (n = 2). The oligodendrogliomas were located in the brainstem and extended into the ventricles, whereas all astrocytomas were located in the forebrain. Isocitrate dehydrogenase 1 gene mutation as described in humans was not detected. The 5 meningiomas exhibited microcystic, chordoid, atypical, papillary, and anaplastic subtypes. Metastatic carcinomas (n = 4) were the only secondary tumor type present, and these were located at the level of the medulla with infiltration of cranial nerves and in one case leptomeningeal carcinomatosis. In addition, 2 medulloblastomas and 1 choroid plexus carcinoma were diagnosed. Immunohistochemistry for vimentin and pancytokeratin was particularly useful to distinguish meningiomas and choroid plexus carcinoma (positive for vimentin only) from mestastatic carcinomas (positive for cytokeratin only) as all showed a papillary growth pattern. Overall, the morphological features were comparable with other species and the human and canine classifications could be applied.

Nervous tissue tumors in cattle are rare and the majority of these are peripheral nerve sheath tumors.^3,16,23^ Tumor surveys in cattle showed that intracranial neoplasms represented only 0.11% to 0.5% of all bovine tumors,^3,6,40,53,56^ which is considerably less than the 2% to 5% reported in dogs.^44^ Other surveys showed that between 0.4% and 4.5% of cattle with neurological signs had intracranial neoplasms.^1,26,27,42^ The types of intracranial tumors reported in cattle include meningioma,^30^ ependymoma,^42^ astrocytoma,^53^ oligodendroglioma,^33^ glioblastoma,^18^ choroid plexus tumor,^26^ medulloblastoma,^45^ malignant peripheral nerve sheath tumor,^64^ neurofibroma,^18^ ganglioneuroblastoma,^49^ pineocytoma (formerly known as pinealoma),^56^ pineoblastoma,^46^ pituitary adenoma,^18^ suprasellar germ cell tumor,^10^ lymphoma,^60^ and metastatic carcinoma.^59^

However, in contrast to the extensive investigation and classification of intracranial tumors in dogs and cats,^28,32^ data for cattle are fragmentary and often based on single case reports. Few individual bovine intracranial tumors have been characterized by immunohistochemistry (IHC).^2,8,10,24,26,31,33,46,64^ The aims of this largest case series of central nervous system (CNS) tumors to date were to describe and characterize 24 bovine intracranial tumors by histology and IHC and to establish if there were any correlations between tumor types and signalment, clinical signs, gross appearance, and/or tumor location.

## Materials and Methods

Twenty-four intracranial neoplasms were identified in cattle in the archive of the Central Veterinary Research Laboratory, Department of Agriculture, Food and the Marine, Backweston, Co. Kildare, Ireland, and of the Pathobiology Section, School of Veterinary Medicine, University College Dublin, Ireland between 1989 and 2019. One of these was previously published as a case report.^55^ Cases represented 0.48% of approximately 4950 cattle that had originally been submitted as clinical suspects for BSE (bovine spongiform encephalopathy) and/or for the investigation of neurological disease during the 30-year period. Data on age, clinical signs, duration of illness, number of tumors, tumor location(s), and gross appearance were reviewed. Clinical signs that had been recorded by veterinary practitioners were grouped into categories as described by Mayhew.^41^

Formalin-fixed, paraffin-embedded brain tissues were available from each case. For histopathological examination, representative coronal sections of the tumor and selected brain regions (mainly cerebral cortex, thalamus, midbrain, medulla at the pons and obex, proximal spinal cord, and cerebellum) were sectioned at 4 μm and stained with Gill-2 hematoxylin and eosin. The intracranial tumors were classified according to the World Health Organization (WHO) international histological classification for humans.^39^ Glial tumors were graded as low- or high-grade using features such as necrosis, microvascular proliferation, mitosis, and universal cellular features of malignancy as described by Koehler et al^34^ for dogs. Meningiomas were graded from grade I to grade III according to the WHO classification for human brain tumors which is based on the 15 different histological tumor subtypes.^39^ Mitotic figures were counted in 10 high-power fields (HPF), equivalent to 2.37 mm^2^ (40× objective and 10× ocular with FN 22 mm, Olympus BX51 microscope, Mason technology, Dublin, Ireland).

Tumor sections of all cases were immunohistochemically labeled for glial fibrillary acidic protein (GFAP), S100, and synaptophysin. Suspect meningioma, choroid plexus tumors, and metastatic cancer were additionally labeled by pancytokeratin (CK) and vimentin. GFAP-negative glial tumors were additionally labeled with oligodendrocyte transcription factor 2 (OLIG2). In addition, on a selection of glial tumors, IHD1 R132H antibody was used for the detection of the mutation in the isocitrate dehydrogenase gene (*IDH1*) commonly seen in humans. Primitive neuroectodermal tumors were additionally labeled with neuron-specific enolase (NSE). The antibodies and protocols are listed in Table 1. Bovine skin (CK, vimentin), brain tissue (GFAP, S100, NSE, OLIG2, synaptophysin), and human brain tissue (IDH1) were used as positive controls. Primary antibody was omitted as negative controls for all established antibodies (CK, vimentin, GFAP, S100, NSE, synaptophysin) and replaced by mouse IgG as negative control for OLIG2. Immunohistochemical results were subjectively categorized based on the number of positively labeled tumor cells: (−), no tumor cells labeled; (+), less than 10% or isolated positive tumor cells; +, 10%–50% of the tumor cells labeled positive; ++, 50%–90% of the tumor cells labeled positive; and +++, greater than 90% of the tumor cells labeled positive as previously reported.^4^ The pattern of labeling, diffuse or patchy, was recorded, as was the part of the cell that was labeled. Furthermore, any positive labeling of stromal, infiltrating, or surrounding cells was recorded.

**Table 1.** Antibodies and protocols used for immunohistochemistry.

Antigen	Species	Clone	Source	Dilution/Incubation Time	Antigen Retrieval
GFAP	Rabbit	Polyclonal	Dako, Gostrup, DK	1:2000; 30 min at 37°C	Tris-EDTA, pH 9.0
S100	Rabbit	Polyclonal	Dako, Gostrup, DK	1:2000; 2 h at 37°C	Tris-EDTA, pH 9.0
Synaptophysin	Mouse	Monoclonal	Dako, Gostrup, DK	1:30; 1.5 h at 37°C	Citrate buffer, pH 6.0
CK	Mouse	Clones AE1/AE3	Dako, Gostrup, DK	1:200; 20 min at RT	Citrate buffer, pH 6.1
Vimentin	Mouse	Clone V9	Dako, Gostrup, DK	1:400; 20 min at RT	Citrate buffer, pH 6.1
OLIG2	Rabbit	Polyclonal	Proteintech, Rosemont, IL, USA	1:500; overnight at 4°C	Tris-EDTA, pH 9.0
NSE	Mouse	Clone BBS/NC/VI-H14	Dako, Gostrup, DK	1:1000; 30 min at RT	Citrate buffer pH 6.0
IDH1 R132H	Mouse	Clone H09	Dianova, Hamburg, DEU	1:100; 30 min at 37°C	Citrate buffer pH 6.0

Abbreviations: GFAP, glial fibrillary acidic protein; CK, pancytokeratin; RT, room temperature; OLIG2, oligodendrocyte transcription factor 2; NSE, neuron-specific enolase; IDH, isocitrate dehydrogenase.

## Results

The age of the cattle affected was recorded in 21 cases and ranged from 0.5 to 14 years (5.7 ± 3.6 years, mean ± SD). Considering the average age of cattle by tumor type (Supplemental Table S1), animals with meningioma appeared to be younger (3.5 ± 0.87, mean ± SD) than animals with oligodendrogliomas (7.7 ± 3.9) and astrocytomas (8.2 ± 1.89). Clinical signs were recorded in 20 cases (Supplemental Table S1). The majority of cattle showed behavioral changes and altered mental state (n = 10), followed by cerebellar incoordination (n = 9) and vestibular syndrome (n = 8). Seizures were not mentioned in any of the animals. The duration of clinical signs in 10 cases ranged from 5 days to 5 months. Two cattle were diagnosed with neurological disease when presented for slaughter.

Gliomas, meningiomas, metastasizing carcinomas, medulloblastomas, and a choroid plexus carcinoma were diagnosed. The anatomical locations of the different tumor types are listed in Supplemental Table S2. Results of IHC are detailed in Table 2.

**Table 2.** Immunohistochemistry for tumor markers in 24 bovine intracranial tumors.

Case No.	Tumor Type	GFAP	S100	Synaptophysin	CK	Vimentin	OLIG2	NSE	IDH1
1	Oligodendroglioma	+	+	−	ND	ND	++	ND	−
2	Oligodendroglioma	+	+	(+)	ND	ND	++	ND	−
3	Oligodendroglioma	+	+	−	ND	ND	++	ND	−
4	Oligodendroglioma	+	+	−	ND	ND	−	ND	−
5	Oligodendroglioma	+	+	(+)	ND	ND	+++	ND	−
6	Oligodendroglioma	(+)	+	−	−	++	ND	ND	ND
7	Astrocytoma	+++	++	−	ND	ND	ND	ND	−
8	Astrocytoma	+++	+++	−	ND	ND	ND	ND	−
9	Astrocytoma	+++	−	ND	ND	ND	ND	ND	ND
10	Astrocytoma	+++	++	−	ND	ND	ND	ND	ND
11	Undefined glioma	+	+	−	ND	ND	++	ND	−
12	Undefined glioma	++	++	(+)	ND	ND	ND	ND	ND
13	Meningioma GRD III	(+)	+	−	−	++	ND	ND	ND
14	Meningioma GRD III	+	(+)	−	−	++	ND	ND	ND
15	Meningioma GRD II	+	(+)	−	(+)	++	ND	ND	ND
16	Meningioma GRD II	+	+	−	(+)	++	ND	ND	ND
17	Meningioma GRD I	−	+	−	+	+++	ND	ND	ND
18	Metastatic carcinoma	−	−	−	+++	−	ND	ND	ND
19	Metastatic carcinoma	−	−	−	+++	−	ND	ND	ND
20	Metastatic carcinoma	+	−	(+)	+++	−	ND	ND	ND
21	Metastatic carcinoma	(+)	(+)	−	+++	−	ND	ND	ND
22	Medulloblastoma	−	+	+	−	++	ND	+	ND
23	Medulloblastoma	+	+	−	ND	ND	ND	++	ND
24	Choroid plexus carcinoma	(+)	ND	ND	−	+++	ND	ND	ND

Abbreviations: GFAP, glial fibrillary acidic protein; CK, pancytokeratin; OLIG2, oligodendrocyte transcription factor 2; NSE, neuron-specific enolase; IDH, isocitrate dehydrogenase; +, 10%–50% of the tumor cells labeled; −, no tumor cells labeled; ND, not done; ++, 50%–90% of the tumor cells labeled; (+), less than 10% or isolated positive tumor cells; +++, greater than 90% of the tumor; GRD, grade.

### Oligodendroglioma

**Figure 1.**
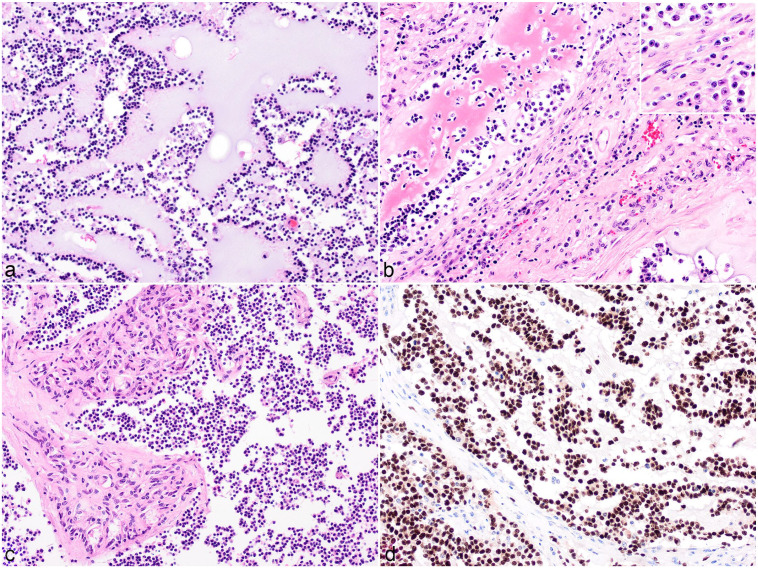
High-grade oligodendroglioma, brain, cattle. (**a)** Large multifocal to coalescing mucin lakes separate dense sheets of small hyperchromatic cells. Case 3. Hematoxylin and eosin (HE). (**b)** Cells show moderate nuclear pleomorphism, larger oval nuclei with fine stippled chromatin, and prominent nucleoli (inset). A focal area of bright eosinophilic amorphous material is consistent with osteoid. Case 5. HE. (**c)** Clusters of blood vessels lined by multiple layers of hypertrophied endothelial cells infiltrate the sheets of small hyperchromatic cells. Case 2. HE. (**d)** Immunohistochemistry for oligodendrocyte transcription factor 2 labels 90% of neoplastic cells with nuclear immunoreactivity. Case 5.

Oligodendroglioma was the most common brain tumor (6/24, 25%; cases 1–6). All infiltrated the medulla, with additional involvement of the cerebellar peduncles in cases 3 and 6. Moreover, soft gelatinous white to gray masses had formed in the fourth ventricle in 5 cases and extended rostrally into the lateral ventricles in cases 3 and 5. Microscopically, the oligodendrogliomas formed well-demarcated densely cellular masses that diffusely infiltrated gray and white matter and occasionally the ependymal layer and leptomeninges. The neoplasms were composed of dense sheets of small round cells (Fig. 1A), which formed occasional pseudorosettes in case 6. Mitotic figures were inconspicuous in 3 cases and ranged between 1 and 15 per 10 HPF in the other 3 cases. These latter cases further showed moderate anisokaryosis, anisocytosis, and nuclear pleomorphism, characterized by larger oval to elongated nuclei that were vesicular or had fine stippled chromatin with prominent and small nucleoli (Fig. 1B). Four tumors showed a bluish-staining myxoid matrix often forming cyst-like lakes (Fig. 1A). Multifocal thick fibrous tissue strands and small islands of microvascular proliferation were seen throughout 5 tumors (Fig. 1C). In case 5, osteoid metaplasia was observed (Fig. 1B). Case 6 had focal marked hyalinization of vascular walls. Multifocal areas of necrosis were observed in 5 tumors often associated with mineralization and occasional cholesterol clefts. In the peripheral neuropil tumor cells aggregated around blood vessels and neurons (secondary structures of Scherer). Based on the above features, all 6 oligodendrogliomas were classified as high grade. Intranuclear immunolabeling for OLIG2 in 70% to 100% of the neoplastic cells was observed in 4/5 cases (Fig. 1D). The tumors were often infiltrated by low numbers of GFAP-positive gemistocytes and the stroma was labeled variably with S100. All 8 gliomas assessed for *IDH1* gene mutation were negative.

### Astrocytoma

**Figure 2.**
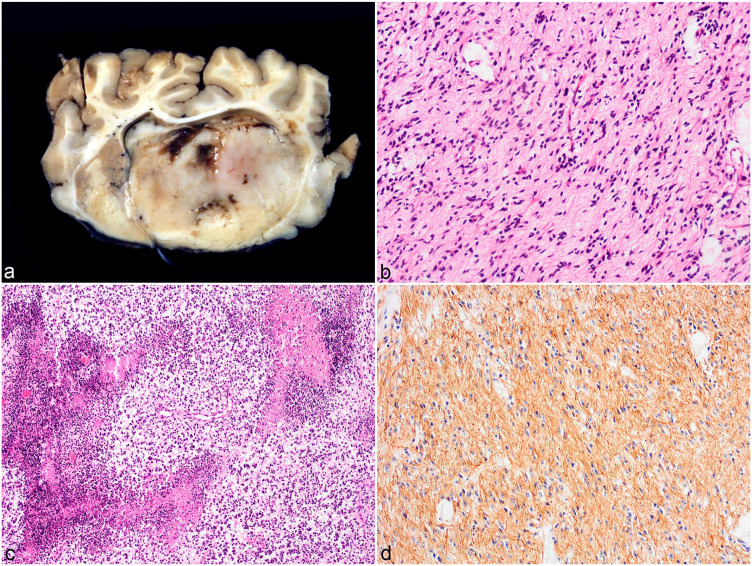
High-grade astrocytoma, brain, cattle. (**a)** Cross section through the cerebrum at the level of the basal ganglion. Infiltrating and expanding the right basal ganglion is a poorly demarcated pale yellow to white mass with multifocal hemorrhages. There is obliteration of the lateral ventricle, midline shift, and compression of the surrounding brain parenchyma. Case 8. (**b)** Fusiform cells with fibrillar eosinophilic cytoplasm and elongated hyperchromatic nuclei are arranged in irregular streams. Case 7. Hematoxylin and eosin (HE). (**c)** Multifocal to coalescing areas of necrosis are surrounded by a densely arranged rim of tumor cells often organized in a perpendicular fashion (pseudopalisading). Case 9. HE. (**d)** Neoplastic fusiform cells have diffuse cytoplasmic immunolabeling for glial fibrillary acidic protein. Case 7.

Astrocytomas infiltrated the brain parenchyma at the level of the basal ganglion and thalamus of 4/24 cattle (16%) (cases 7–10) (Fig. 2A) and extended into the midbrain in 2 of these. Microscopically, astrocytomas were poorly demarcated, moderately to densely cellular masses composed of fusiform, spindle-shaped, and occasionally polygonal cells, arranged in sheets or irregular streams in a fine fibrovascular or preexisting neuropil stroma (Fig. 2B). The cells had indistinct cell borders and moderate to abundant fibrillar and occasionally vacuolated eosinophilic cytoplasm. The nuclei were round to oval and occasionally hyperchromatic but mainly with coarsely stippled chromatin, prominent small nucleoli, and moderate anisokaryosis and anisocytosis. In 3 tumors, the mitotic count ranged from 1 to 15 per 10 HPF. Multifocal to coalescing areas of necrosis were seen in these 3 cases often surrounded by dense clusters of neoplastic cells (Fig. 2C). There were multifocal prominent capillaries and proliferation of blood vessels arranged in dense clusters surrounded by fibrous tissue in all 4 tumors. Based on the above features, all astrocytomas were classified as high grade. Diffuse cytoplasmic brown immunolabeling of neoplastic cells for GFAP was observed in all 4 cases (Fig. 2D).

### Undefined Glioma

**Figures 3.**
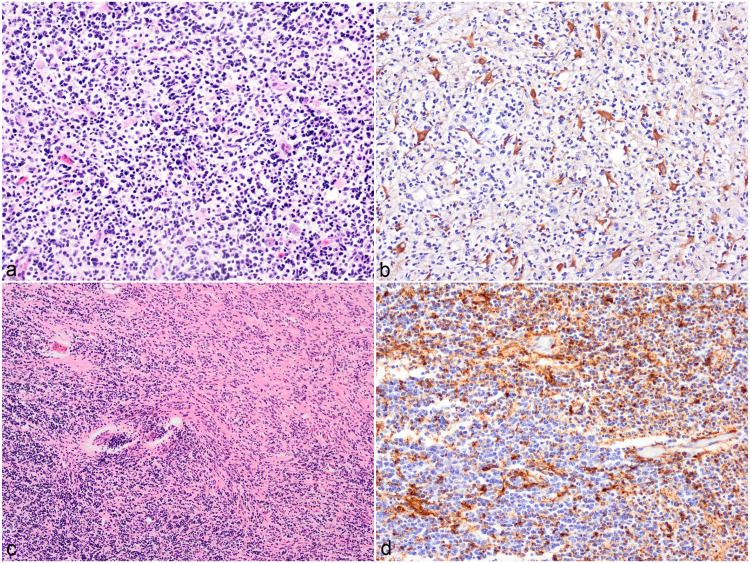
Undefined glioma, brain, cattle. (**a)** Sheets of small hyperchromatic cells (oligodendrocyte-like cells) are admixed with numerous large angular cells with abundant eosinophilic cytoplasm (gemistocyte-like cells). Case 11. Hematoxylin and eosin (HE). (**b)** Gemistocyte-like cells show cytoplasmic immunolabeling with glial fibrillary acidic protein (GFAP). (**c)** There is collision of 2 cell populations: sheets of small hyperchromatic cells (oligodendrocyte-like cells) and streams of fibrillar fusiform cells (astrocyte-like cells). Case 12. HE. (**d)** Immunohistochemistry for GFAP shows brown immunolabeling of the fusiform cell population.

The 2 undefined glial tumors (cases 11 and 12) were composed of 2 distinct neoplastic cell populations resembling oligodendrocytes and astrocytes. In case 11, the tumor infiltrated the cerebellar white matter and peduncle. It showed a diffuse growth pattern comprising dense sheets of about 70% OLIG2-positive oligodendroglial cells admixed with 30% large GFAP-positive gemistocyte-like cells (Fig. 3A, B). Case 12 showed a collision growth pattern in a mass infiltrating the basal ganglion. The spindle-shaped and fusiform cells (astrocytes, GFAP-positive) were arranged in streams and bundles in a fibrovascular stroma and the polygonal cells (oligodendrocytes, GFAP-negative) were in sheets within a well-vascularized stroma with occasional fibrous tissue strands. At the border between the 2 areas, there appeared to be a mix of the 2 cell types (Fig. 3C, D). Both tumors were categorized as high grade based on the mitotic count (4 and >20 per 10 HPF, respectively), moderate to marked anisocytosis and anisokaryosis, and the presence of multinucleated cells, necrosis, and vascular proliferation.

### Meningioma

**Figure 4.**
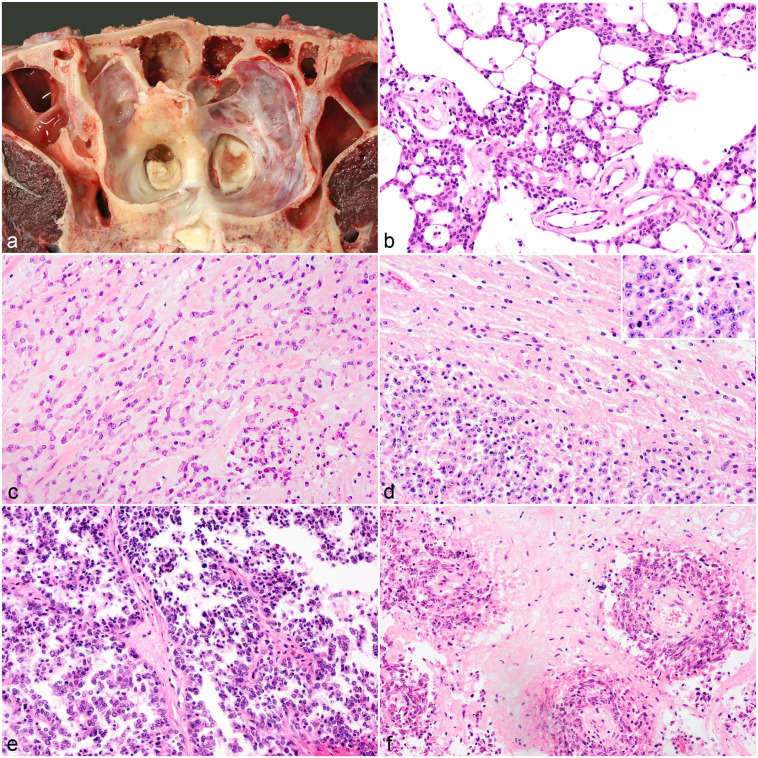
Meningioma, brain, cattle. (**a-b)** Microcystic meningioma, meninges, cow, Case 17. (**a)** A yellow circumferential mass in the dura mater narrows the left frontal and ethmoid fossa and causes mild hyperostosis of the frontal bone. (**b)** Nests of epithelioid cells with occasional intracellular small vacuoles are separated by variably sized interstitial empty cysts in a well-vascularized stroma. Hematoxylin and eosin (HE). (**c)** Chordoid meningioma. Cords of epithelioid cells separated by pale basophilic (myxoid) and bright eosinophilic matrix. Case 15. HE. (**d)** Atypical meningioma, midbrain. Sheets of polygonal cells infiltrate into the brain parenchyma and have moderate to scant eosinophilic cytoplasm, vesicular oval nuclei, and frequent mitotic figures (inset). Case 16. HE. (**e)** Papillary meningioma. Single to multiple layers of polygonal cells line fibrovascular tissue stalks. Case 14. HE. (**f)** Anaplastic meningioma, right cerebral hemisphere. Spindle-shaped and polygonal cells form whorls around blood vessels when infiltrating the brain parenchyma. Case 13. HE.

Meningioma was the second most common intracranial tumor (5/24, 21%). Of these, 4 were in the forebrain region including meninges and the lateral ventricle, and 1 was present at the level of the midbrain. Each of these represented a different histologic subtype with 1 grade I, 2 grade II, and 2 grade III tumors. In case 17, the neoplasm was confined to the dura mater and leptomeninges overlying the left olfactory and frontal lobes and appeared as soft yellow granular thickening with moderate hyperostosis of the underlying bones (Fig. 4A) that narrowed the left frontal and ethmoid fossa and corresponded with a depression in the left frontal cortex. There was marked hydrocephalus of the lateral ventricles. Microscopically, infiltrating the meninges were polygonal cells forming nests and islands. The cells had indistinct cell borders and moderate eosinophilic cytoplasm which was occasionally vacuolated. Small oval nuclei with coarse stippled chromatin and occasional prominent small nucleolus were evident. There was mild anisocytosis and anisokaryosis and 1 mitotic figure per 10 HPF. The nests and islands were separated by a net-like structure composed of fine fibrous tissue strands, variably sized empty vacuoles, and collagen-rich often-hyalinized blood vessels (Fig. 4B). Multifocal small areas of necrosis were observed with cells palisading around it. Based on these findings, a diagnosis of microcystic meningioma was made.

Case 15 was a densely cellular infiltrative mass consisting of polygonal epithelioid cells arranged in cords or sheets in a myxoid or osteoid matrix (Fig. 4C). The cells had mainly indistinct cell borders, moderate eosinophilic cytoplasm, and large oval vesicular nuclei with occasional small nucleoli, and 15 mitotic figures per 10 HPF. Multifocal mild hemorrhage, multifocal necrosis, and areas of calcification were observed. It was diagnosed as a chordoid meningioma.

In case 16, the brain parenchyma was infiltrated by dense sheets of polygonal epithelioid cells (Fig. 4D) with scant cytoplasm and large nuclei and 10 mitotic figures per 10 HPF. A diagnosis of atypical meningioma was made.

Case 14 had an infiltrative densely cellular mass characterized by polygonal to spindle-shaped cells arranged in sheets or palisades along fibrous tissue cores that contained blood vessels arranged in papilla-like structures (Fig. 4E). Occasionally, tumor cells palisaded around blood vessels forming pseudorosettes. The spindle-shaped cells had moderate eosinophilic fibrillar cytoplasm, whereas the cuboidal cells had distinct to indistinct cell borders and moderate to scant eosinophilic cytoplasm. The nuclei ranged from elongate, to oval or round with finely stippled chromatin, to vesicular with prominent small nucleoli. The mitotic rate was high with >25 mitotic figures per 10 HPF and there was moderate anisocytosis and anisokaryosis. There were multifocal to coalescing large areas of necrosis and hemorrhage within the mass. Multiple areas of osseous metaplasia were present. Based on the above features, a diagnosis of papillary meningioma was made.

In case 13, the mass was composed of spindle-shaped cells arranged with variable density in short bundles and whorls around blood vessels (Fig. 4F). In other areas cells were more polygonal and formed pseudorosettes. Marked anisocytosis and anisokaryosis and scattered multinucleated cells were present. Thirty mitotic figures were seen per 10 HPF. The stroma consisted of thick fibrous tissue often hyalinized with occasional bone metaplasia. Multifocal marked areas of necrosis often associated with calcification and small calcified bodies were observed. Based on the high mitotic rate and mixed papillary to sarcomatous pattern, the mass was diagnosed as an anaplastic meningioma. Overall, most neoplastic cells labeled positive for vimentin and only the grade I tumor showed some CK expression.

### Metastatic Carcinoma

Metastatic carcinomas of unknown primary locations were observed in 4 cases (cases 18–21) at the level of the medulla. The diagnosis was based on the epithelioid characteristics of the neoplastic cells and immunolabeling for CK only.

**Figure 5.**
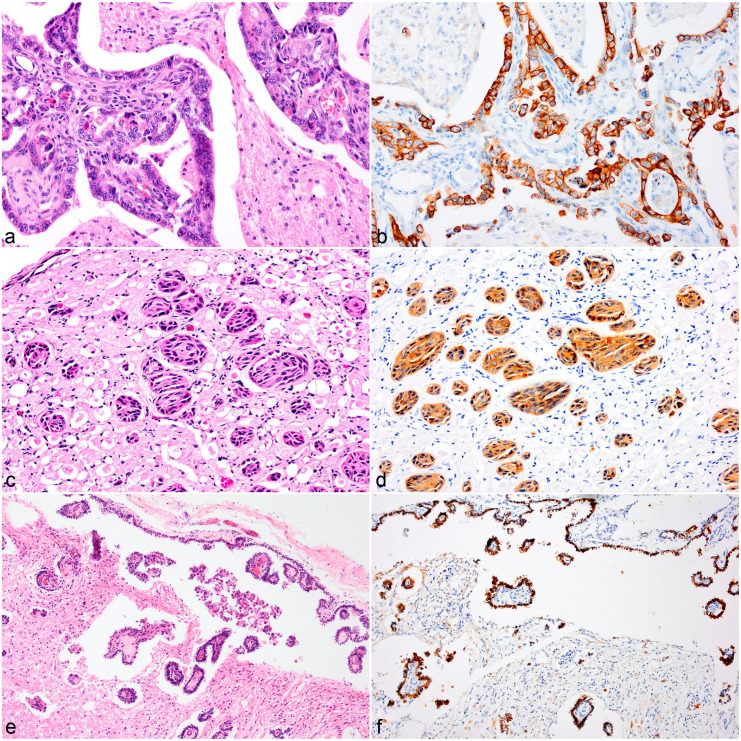
Metastatic carcinoma, brain, cattle. (**a-b)** Medulla oblongata, case 20. (**a)** Fibrovascular tissue cores lined by single to multiple layers of cuboidal cells infiltrate the gray matter. Hematoxylin and eosin (HE). (**B)** Cytoplasmic immunolabeling of neoplastic cells with pancytokeratin (panCK). (**c-d)** Oculomotor nerve, case 19. (**c)** Variably sized nests of polygonal cells infiltrate the nerve fibers. HE. (**d)** Immunohistochemistry for panCK labels the cytoplasm of infiltrating epithelioid cells. (**e-f)** Meninges at the level of the medulla oblongata, case 18. (**e)** Cuboidal cells line the subarachnoid space and extend along the perivascular spaces. HE. (**f)** Cytoplasmic immunoreactivity for cuboidal cells with panCK.

Two of these tumors (cases 20 and 21) were poorly demarcated unencapsulated papillary masses extending from the meninges via the trigeminal nerve into the medulla. These were composed of papillary projections with fibrous tissue cores lined by single to multiple layers of cuboidal cells with occasional multinucleated cells. These papillae often formed cystic spaces some of which were filled with necrotic cell debris (Fig. 5A, B).

In case 19, islands of squamous epithelial cells infiltrated the oculomotor nerve (Fig. 5C, D) and filled the perivascular spaces of the ventral midbrain and medulla.

A third type was seen in case 18, where cuboidal to columnar cells mainly in single layers lined the subarachnoid space surrounding the brain stem, formed small papillae, or palisaded around blood vessels in the perivascular spaces in the brainstem (Fig. 5E, F). Marked anisocytosis and anisokaryosis with occasional binucleate or multinucleate cells were seen. The carcinomas had 2 to 5 mitotic figures per 10 HPF.

### Medulloblastoma

**Figure 6.**
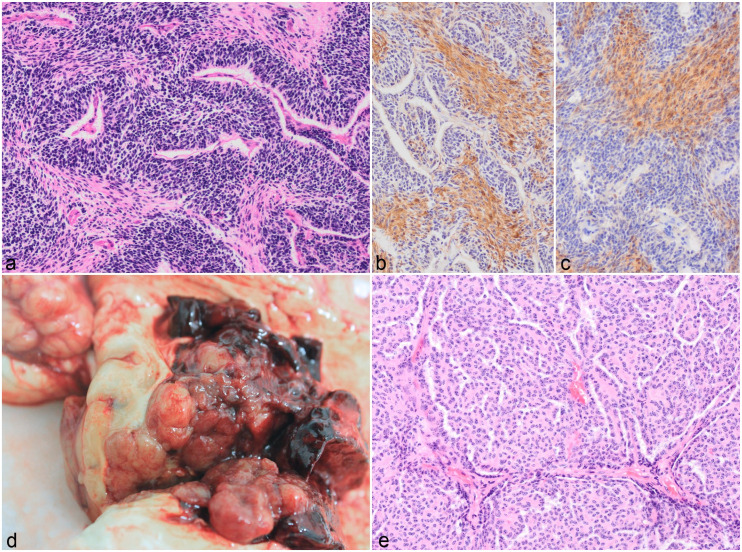
**(a–c)** Medulloblastoma, cerebellum, heifer, case 23. **(a)** Fusiform cells with hyperchromatic nuclei are densely arranged and form palisades (pseudorosettes) up to 7 cells thick around blood vessels and are separated by streams of eosinophilic spindle-shaped cells. Hematoxylin and eosin (HE). The eosinophilic spindle-cell population is immunolabelled for S100 (**b**) and neuron-specific enolase (**c**). **(d-e)** Choroid plexus carcinoma, lateral ventricle, cow, case 24. **(d)** A multilobulated yellow mass surrounded by hemorrhage expands the lateral ventricle and infiltrates the cerebrum. **(e)** Cuboidal cells form branching chords and trabeculae with occasional fibrovascular tissue cores. Hematoxylin and eosin.

The 2 cases of medulloblastoma (cases 22 and 23) were located in the cerebellum. The tumors were characterized by densely arranged fusiform to polygonal cells in a laminar pattern forming pseudorosettes or rare true rosettes. These cells had hyperchromatic nuclei. These areas were separated by a second cell population arranged in streams that consisted of spindle-shaped cells with abundant eosinophilic fibrillar cytoplasm (Fig. 6A) and 4 to 8 mitotic figures were seen per 10 HPF. Multifocal small areas of necrosis with calcification were observed throughout. Only the spindle-shaped cells labeled with S100 and NSE (Fig. 6B, C).

### Choroid Plexus Carcinoma

Case 24 was a multinodular, well-demarcated, light brown to dark red, hemorrhagic mass that expanded the right lateral ventricle, extended into the left lateral ventricle, and infiltrated small areas of the right parietal cerebral cortex (Fig. 6D). It was composed of cuboidal cells arranged in an arboriform papillated pattern (Fig. 6E) or occasionally solid pattern. Moderate cellular pleomorphism and 5 mitotic figures per 10 HPF were observed. There were large areas of hemorrhage and necrosis within the tumor. The neoplastic cells were positive for vimentin only. Based on the above features, a diagnosis of choroid plexus carcinoma was made.

## Discussion

The present study is the largest comprehensive survey conducted on bovine intracranial brain tumors to date and several tumor subtypes are described for the first time in this species. The prevalence was very low, estimated at 0.48%, which is similar to one previous report.^26^ Despite their rarity, these tumors were comparable with other species in their histologic patterns, IHC labeling, and tumor types and grades.^28,34,39,46^

Overall, 4 different primary brain tumors were diagnosed: glioma, meningioma, medulloblastoma, and choroid plexus tumor. Carcinoma was the only secondary brain tumor. The most common tumors were gliomas and meningiomas, which is similar to reports in dogs.^57,58^ Secondary brain tumors comprised 17% of the cases in this study, which is considerably lower than in dogs (about 50%)^57^ and cats (29%).^61^

Intracranial neoplasms occurred in cattle at any age with medulloblastomas being particularly present in juvenile animals (6 and 18 months). Medulloblastomas are one of the more commonly reported brain tumors in cattle generally affecting calves from stillborn up to 4 months of age.^2,8,29,45^ Meningiomas were diagnosed in this report in adult cattle with a lower mean age than animals with oligodendrogliomas or astrocytomas. This trend has been described in the literature where the majority of cattle with meningiomas were ≤2 years of age.^17,30^

The main clinical manifestations of the intracranial neoplasms in the present study were altered mental stage, central vestibular dysfunction, and cerebellar incoordination. These were nonspecific signs and likely related to the location of the mass lesion and the associated increased intracranial pressure. The most frequent clinical signs in dogs with brain tumors are seizures.^57^ These were not observed in the present case series, as cattle have a relatively high seizure threshold and the main causes for seizures in this species are metabolic disturbances or marked neuronal damage.^15^

Most tumor types reported here had a distinct anatomical location. All oligodendrogliomas infiltrated the brainstem at the level of the medulla and commonly extended as large masses into the ventricular system. This contrasts with the 2 case reports on oligodendrogliomas in cattle where the neoplastic cells diffusely infiltrated the leptomeninges from the midbrain to the cerebrum^31,33^ and formed a nodular mass on the dorsal aspect of the thalamus.^31^ In dogs oligodendrogliomas are typically found in the frontal, parietal, and temporal lobes.^63^ Astrocytomas, on the contrary, arose in this case series supratentorially at the level of the basal ganglia and thalamus and extended into the midbrain in 2 cases, which is similar to what has been described in 3 bovine case reports^50,53,64^ and for other species.^63^ The meningiomas were mainly located over the cerebral hemispheres; only the midbrain was affected in one case. This is in accordance with previous reports on 4 out of 5 bovine meningiomas that were seen at the level of the cerebrum.^17,30^ The metastatic carcinomas infiltrated the lateral medulla at the level of the pons in all cases and the ventral midbrain and the subarachnoid space each in one of these cases. Three case reports on metastasizing squamous cell carcinomas in cattle found a similar involvement of the lateral brainstem and ventral midbrain.^51,59,65^ Medulloblastomas are of embryonal origin and arise in the cerebellum^29^ as seen in this study. The choroid plexus tumor in this series was found in the lateral ventricle, whereas it has been reported in cattle in the third and fourth ventricles previously.^24,50,64^ In dogs it can arise in any ventricle but was most commonly seen in the fourth.^11,14^

Glial tumors encompassed 50% of the intracranial neoplasms in cattle in the current study. All glial tumors were high grade based on the revised diagnostic classification of canine gliomas.^34^ In a recent prognostic study on canine glial tumors, the histologic features of high-grade gliomas correlated with poor survival.^43^ Previous case reports in cattle also described high-grade glial tumors.^31,33,64^ One tumor classified as of oligodendroglial origin based on the mucinous matrix, absence of GFAP labeling, OLIG2 labeling of more than 90% of neoplastic cells, and typical cell phenotype in most areas contained osteoid metaplasia. This is a rare finding in glial tumors and is mainly associated with gliosarcomas.^62^

Differentiation of astrocytomas and oligodendrogliomas was based on the cellular morphology and growth pattern; however, the typical “honeycomb pattern” characteristic of well-differentiated oligodendrogliomas described in dogs^20,34^ was not observed. IHC for GFAP helped in the identification of astrocytes. OLIG2 is a CNS-restricted transcription factor that plays a critical role in glial progenitor proliferation^36^ and is ubiquitously expressed in gliomas.^20,28,38^ It can be used as a prognostic indicator as loss of expression has been linked to lower survival in humans.^9^ While the antibody for OLIG2 worked well on bovine brain tissue, only 4 out of 5 oligodendrogliomas showed the distinct nuclear immunolabeling. This may have been due to loss of expression, or secondary to advanced autolysis or overfixation.^34^ In humans, classification of gliomas is now based on the presence or absence of a shared genetic mutation in the *IDH1* gene and this key genetic alteration characterizes gliomas with favorable outcome.^12,39^ Diffusely infiltrating gliomas including glioblastomas are grouped together^39^ and IHC with an antibody targeting the single-point mutation IDH1 R132H is used for the initial diagnosis.^12^ The *IDH1* gene is highly conserved, and bovine and human sequences are identical in the area that is targeted by the antibody. The lack of immunolabeling in 8 of the bovine gliomas examined may suggest a different underlying carcinogenesis; however, further validation of the antibody in cattle is necessary to support this assumption.

The 5 meningiomas described in this study represented 5 of the 15 different subtypes described for humans,^39^ thereby showing pathological similarities with human meningiomas as has also been reported in dogs.^47^ However, benign meningiomas, diagnosed only in 20% of the cases in this report, occurred less frequently than has been reported in humans (80%)^39^ and in dogs (around 50%).^47^ Infrequent cases of meningioma have been described in cattle,^1,13,17,30,48^ and apart from an anaplastic meningioma,^48^ none of the meningeal subtypes encountered here have been reported before. The majority has been described as the fibroblastic subtype and a link with bovine papillomavirus infection has been shown experimentally.^21^ While most meningiomas grow as well-demarcated masses,^47^ the microcystic meningioma did not form a distinct mass but led to diffuse thickening of the meninges circumferentially surrounding and compressing the olfactory lobe and cranial frontal cortex. This was associated with hyperostosis which is rarely reported in dogs but common in cats.^47^ The IHC results reported here are similar to what has been observed in dogs.^28,54^

In the current study, IHC for vimentin and CK was particularly useful in distinguishing the papillated/anaplastic meningiomas and choroid plexus tumor from carcinomas, as these all exhibited a papillary growth pattern.^52^ Primary carcinomas in the brain are extremely rare and are suspected to arise from intracranial epidermoid or dermoid cysts.^37^ As no cysts were seen in any of the cases and have not been reported in cattle in the literature, it is presumed that these carcinomas represent secondary metastases. The infiltrations of cranial nerves in 3 of the cases and subarachnoid spread in the fourth case indicate perineural invasion and spread of the neoplasm along the cranial nerves to the brain.^5^ In humans, perineural invasion commonly occurs with squamous cell carcinomas around the head and neck^5^ and has been suggested for cattle.^59^ Metastatic carcinomas have a diverse histopathology^52^ as can be observed in the present study, where histopathologic features ranged from papillae and small nests to widespread meningeal infiltration resembling leptomeningeal carcinomatosis. Typical features of squamous differentiation such as keratin pearls and desmosomes as described in previous case reports^51,59,65^ were not observed. Most of the previous reports of intracranial squamous cell carcinoma in cattle found a link to a squamous cell carcinoma in and around the eye and maxillary sinus.^51,59,65^ Unfortunately, too little history was provided with the cases in this study and the presence of possible primary tumors is unknown. While the rate of secondary brain tumors in cattle is lower than in cats and dogs,^19,61^ secondary carcinomas are infrequent in these species and metastases are mainly located in the cerebrum suggesting hematogenous spread.^57,61^

Choroid plexus carcinomas also show a papillary growth pattern^52^ as was observed in the present study. The neoplastic cells expressed vimentin but not cytokeratin, thereby clearly distinguishing it from a metastatic carcinoma. Choroid plexus tumors in dogs and cattle have been reported to consistently express vimentin but to variably label with cytokeratin.^11,24–26,64^ The choroid plexus carcinoma was distinguished from an ependymoma, which has been reported in cattle,^6,40^ by the lack of GFAP expression and absence of typical morphological features such as rosette and pseudorosette formation.^63^ The antibody Kir7.1, inward rectifier potassium channel, specific to choroid plexus cells,^22^ has been shown to work in dogs^14^ and may be useful in cattle as well if a diagnosis based on immunophenotyoing using CK and GFAP and morphology cannot be reached.

Medulloblastomas are thought to arise in the external germinal cell layer of the cerebellum.^35^ Diagnosis was based on the typical dense hyperchromatic fusiform to polygonal cells arranged in a laminar pattern and the presence of pseudorosettes and rosettes within the cerebellum. Neoplastic cells have been reported to variably express neuronal and glial markers.^28^ Case reports in calves showed a lack of GFAP expression,^2^ diffuse positivity for the neuronal markers NSE and synaptophysin, and multifocal labeling for S100.^8^ In the cases in this study, only the spindle-shaped cells showed positivity for NSE and S100, which supported the diagnosis of medulloblastoma. In humans, different histological types have been reported which in combination with 4 different genetic variants are now used as a clinically relevant integrated classification.^39^

This study highlights morphological similarities between brain tumors in cattle and in other species. The data further show trends in age and distribution for tumor types which will support pathologists in making a diagnosis of these rare entities. A recent study on the inter- and intra-observer agreement of canine and feline nervous tissue tumors highlighted again the importance of morphology rather than the use of special stains and IHC for diagnostic accuracy.^7^ In this case series, IHC was found useful primarily for the differentiation between epithelial and other papillary tumors and for the identification of glial cells.

## Supplemental Material

sj-pdf-1-vet-10.1177_03009858221100433 – Supplemental material for Bovine intracranial neoplasia: A retrospective case series Click here for additional data file. Supplemental material, sj-pdf-1-vet-10.1177_03009858221100433 for Bovine intracranial neoplasia: A retrospective case series by Hanne Jahns and Maire C. McElroy in Veterinary Pathology
