# Supplementary material for: Bovine intracranial neoplasia: A retrospective case series
Source: Vet Pathol. 2022 May 31;59(5):824–35. doi: 10.1177/03009858221100433 (PMC9358308; doi:10.1177/03009858221100433)
Supplement: sj-pdf-1-vet-10.1177_03009858221100433 – Supplemental material for Bovine intracranial neoplasia: A retrospective case series [file sj-pdf-1-vet-10.1177_03009858221100433.pdf]

Supplemental Table S1; The age (n = 21), duration of illness (n = 10) and predominant clinical signs (n = 20) are listed according to the bovine intracranial tumor types.

Tumor type (n)	Age (years)	Median duration of clinical signs (days)	Behavioral changes/ Altered mental state	Opistho tonus/ tremor	Cranial nerve deficits	Vesti bular signs	Cerebellar incoordina tion	Paresis
Oligodendro glioma (6)	7.7 ± 3.9	8.5 (range 7-10)	4	0	0	2	2	1
Astrocytoma (3)	8.2 ± 1.89	21 (range 5-90)	1	0	0	0	1	1
Undefined gliomas (2)	1	NR	1	0	0	2	2	0
Meningioma (3)	3.5 ± 0.87	28 (range 14-42)	1	1	1	1	0	2
Metastasizing carcinoma (4)	5.3 ± 1.64	77.5 (range 5-150)	1	0	1	2	3	0
Medulloblasto ma (1)	1 ± 0.7	14	1	0	0	1	1	0
Choroid plexus carcinoma (1)	11	NR	1	0	0	0	0	0
All tumors	5.7 ± 3.6	14 (range 5-150)	10	1	2	8	9	4

NR not recorded

Supplemental Table S2; Anatomical location(s) for 24 intracranial bovine tumors

Tumor type (n)	Cerebral cortex	Basal ganglion/ Thalamus	Midbrain	Brainstem	Cerebellum	Intra ventricular
Oligodendro glioma (6)	0	0	0	6	2	5
Astrocytoma (4)	0	4	2	0	0	0
Undefined glioma (2)	0	1	0	0	1	0
Meningioma (5)	3	1	1	0	0	1
Metastatic carcinoma (4)	0	0	1	4	0	1
Medulloblastoma (2)	0	0	0	0	2	0
Choroid plexus carcinoma (1)	0	0	0	0	0	1
